# Effects of immunotherapy on mortality in neonates with suspected or proven sepsis: a systematic review and network meta-analysis

**DOI:** 10.1186/s12887-019-1609-1

**Published:** 2019-08-05

**Authors:** Yuhang Li, Shulong Yang, Guiyue Wang, Miao Liu, Zhaodi Zhang, Haitao Liu, Kaijiang Yu, Changsong Wang

**Affiliations:** 1 Department of Critical Care Medicine, Harbin Medical University Cancer Hospital, No. 150 Haping Rd., Nangang District, Harbin, 150081 China; 2 Department of Anesthesiology, The First Affiliated Hospital Sun Yat-sen University, Guangzhou, China; 3 Department of Pediatric surgery, the Second Affiliated Hospital of Harbin Medical University, Harbin, China; 4 Department of Anesthesiology, Tianjin Medical University Cancer Hospital, Tianjin, China; 5 Department of Anesthesiology, Xuanwu Hospital Capital Medical University, Beijing, China; 6 Department of Anesthesiology, Harbin Medical University Cancer Hospital, Harbin, China

**Keywords:** Neonate, Sepsis, Immunotherapy, Mortality, Meta-analysis

## Abstract

**Background:**

To investigate the efficacies of different immunotherapies in neonates with suspected or proven sepsis.

**Methods:**

We searched the Cochrane Library, EMBASE, MEDLINE, EBSCOhost, and Web of Science for studies published before May 2019 that investigated different immunotherapies in neonates with suspected or proven sepsis. Comparisons were among immunotherapies and between immunotherapy and placebo. The review was registered in the PROSPERO CRD database.

**Results:**

All-cause mortality was not significantly different between patients who received the immunoglobulin (IgG), IgM-enriched immunoglobulin (IgGAM), granulocyte-colony stimulating factor (G-CSF) or granulocyte-macrophage colony stimulating factor (GM-CSF) immunotherapies and those who received placebo. The RRs of the immunotherapies were 0.80 (95% CI: 0.57 to 1.1), 0.45 (95% CI: 0.17 to 1.0), 0.93 (95% CI: 0.64 to 1.2) and 0.67 (95% CI: 0.39 to 1.1), respectively. Compared with placebo, none of the interventions showed statistically significant differences in the duration of hospital stay. The MDs of the immunotherapies were − 2.7 (95% CI: − 8.4 to 3.5), − 0.18 (95% CI: − 7.3 to 7.7), − 1.7 (95% CI: − 7.3 to 3.9) and − 7.2 (95% CI: − 28 to 13), respectively.

**Conclusions:**

No significant differences in all-cause mortality or the duration of hospital stay were found in neonates with suspected or proven sepsis treated with the four types of immunotherapies and those treated with placebo.

**Electronic supplementary material:**

The online version of this article (10.1186/s12887-019-1609-1) contains supplementary material, which is available to authorized users.

## Background

Neonatal sepsis is a major cause of neonatal mortality worldwide, accounting for approximately 1.4 million neonatal deaths annually [1]. Despite numerous advances in neonatal intensive care, neonatal sepsis remains an important cause of mortality and morbidity in infants, as improving accuracy in the diagnosis and treatment of neonatal sepsis has been challenging [2].

Neonatal sepsis varies markedly from sepsis in adults. Despite years of clinical experience, challenges in the treatment of neonates with suspected or proven sepsis, including the lack of a consensus definition [3]. Routine treatment of neonatal infection includes antimicrobial therapy for the suspected or proven pathogens, and differences in time presentations and exposure affect the choice of antimicrobial agents. The most important components for determining which treatment to use are a complete medical history, physical examination and cultures of clinical specimens. Empirical therapy is usually guided by the antimicrobial resistance patterns of bacteria detected in the neonatal intensive care unit and community settings. Once the pathogens have been identified, the most appropriate antimicrobials should be used [4]. However, these therapies may not be equally effective for all patients, particularly those with severe comorbidities or difficult-to-treat infections. Thus, new patient-tailored therapies are required. Additionally, immune dysfunction or suppression is increasingly being recognized as a critical factor in sepsis.

The immune system is underdeveloped in neonates. The neonate usually relies on an immature innate immune system [5], and maturity may be linked to the developmental age of the neonate. Preterm infants are at the greatest risk of developing sepsis [6]. Despite their dependence upon innate immunity, neonates have a deficient innate response to infection, which further increases their risk of further bacterial, fungal, and viral infections [7, 8]. The modulation of the neonatal immune system to reduce the sepsis mortality, and sepsis survivor morbidity would be a great advance in the field.

The role of immunotherapy in augmenting the immature immune system has been extensively studied. Different types of immunomodulatory agents, such as immunoglobulin (IgG), IgM-enriched immunoglobulin (IgGAM), granulocyte-colony stimulating factor (G-CSF), granulocyte-macrophage colony stimulating factor (GM-CSF) and human antibodies to endotoxin (antilipopolysaccharide), have been evaluated for use in treating neonatal sepsis. Polyvalent IgG has been shown to improve opsonization, prevent nonspecific complement activation, and neutralize endotoxin [9, 10]. IgGAM has been shown to improve antibacterial activity [11, 12]. G-CSF stimulates myeloid progenitor cell proliferation and increases the bone marrow storage pool and the number of circulating mature neutrophils [13, 14]. GM-CSF stimulates the production and antibacterial function of neutrophils and monocytes [15, 16]. However, there is no definitive evidence regarding which type of immunotherapy is most effective. A large multicenter randomized clinical trial showed that treatment with intravenous immunoglobulin (IVIg) reduced the early mortality rate but did not significantly affect the overall survival rate in septic neonate patients [17]. Another multicenter randomized clinical trial showed that all-cause mortality decreased among preterm neonates with sepsis and neutropenia who were treated with G-CSF adjunctive therapy [18]. The results of previous systematic reviews and meta-analyses indicated that intravenous immunoglobulin therapies had a positive effect on reducing mortality from neonatal sepsis [19]. Another recent meta-analysis that included more trials demonstrated that intravenous immunoglobulin therapies showed no benefit regarding mortality among neonates with sepsis [20]. These studies were conventional meta-analyses that did not investigate the efficacies of different types of immunotherapies in neonates with sepsis. Moreover, consistent results have not been reported, and none of the studies have definitively established whether immunotherapies offer clinically important benefits for neonatal sepsis.

The purpose of this study was to conduct a network meta-analysis to identify the specific types of immunotherapies that are most effective for neonates with sepsis.

## Methods

We followed the Preferred Reporting Items for Systematic Reviews and Meta-Analyses (PRISMA) guidelines to perform our systematic review [21]. The study protocol for this meta-analysis was registered in PROSPERO (CRD42017080873).

### Data sources and searches

We searched the Cochrane Library, Medline, EMBASE, CINAHL and Web of Science databases from database establishment to May 2019. We used a combination of Medical Subject Headings (MeSH) and text words related to ‘immunotherapy’, ‘G-CSF’, ‘GM-CSF’, ‘IgG’ ‘IgGAM’, ‘sepsis’, ‘septic shock’, ‘neonate’, ‘neonatal’, ‘infant, newborn’ and ‘randomized controlled trials’. The detailed search strategy is shown in electronic Additional file 1: Text E1. In addition, we placed no restrictions on language or year of publication. We also manually searched the database for randomized controlled trials (RCTs) and meta-analyses that may have been missed in the initial electronic search.

### Literature inclusion and exclusion criteria and quality assessment

Two groups of authors developed the search strategy and searched the databases. Three authors (YHL, GYW and ML) independently screened studies based on the title and abstract obtained from the database. Then, two other groups of authors read the selected full texts of the selected articles, assessed all trials for eligibility and extracted relevant information using a predefined data extraction form. Any disagreements were resolved by KJY.

The present study included all RCTs comparing immunotherapies to placebo in patients with sepsis and septic shock that were published before May 2019. The included patients were neonates, whom are infants within the first 28 days after birth. The diagnosis of neonatal sepsis relied on subjective interpretation due to the lack of specific routine laboratory tests [22, 23]. Even the gold standard test, blood cultures, yields positive results in fewer than 10% of cases of suspected sepsis [24, 25]. Suspected infection was usually based on clinical symptoms and signs consistent with infection, without the identification of a causative organism. Proven infection was usually based on clinical symptoms and signs consistent with infection, in addition to the identification of a causative organism. The control groups received either no treatment or placebo (albumin or normal saline). Randomized clinical trials reporting clinical outcomes that compared specific types of immunotherapies with placebo were included. We excluded studies that included patients older than 28 days after birth as well as reviews, retrospective studies, observational studies, case reports, animal studies, irrelevant studies and duplicate studies.

### Outcome measures and data extraction

The extracted data included basic study information, such as experimental design, experimental time, country of the study, inclusion criteria, age and gender of the included patients, detailed experimental interventions, and clinical and safety outcomes for patients with sepsis.

The interventions in the included studies were immunotherapies used to treat suspected or proven neonatal sepsis. Four immunotherapies were analyzed: G-CSF, GM-CSF, IgG and IgGAM. The effectiveness of different immunotherapies was evaluated by comparing them with the effects of placebo. Comparisons were made between immunotherapies and placebo and among the four immunotherapies.

The primary outcome of this study was all-cause mortality in neonates with sepsis. The secondary outcomes of this study included the durations of intensive care unit (ICU) stay, mechanical ventilation, and hospital stay. Two groups of authors separately extracted the data, and the data were subsequently compared and verified.

We excluded RCTs if they did not provide sufficient information with which to judge their eligibility criteria or relevant outcomes. We divided the immunotherapies into four groups and placebo based on their therapeutic agent to compare the effects of all immunotherapy agents that have been clinically studied to date. A type of human antibody to endotoxin was used on neonates with sepsis and low birthweight [26]; however, at present, only one study has reported on this type of immunotherapy. Thus, we did not evaluate this immunotherapy in the present study.

We assessed the risk of bias in the included trials using the Cochrane risk of bias tool. None of the selected publications were excluded based on research quality.

### Statistical analysis

Statistical analyses were performed within a Bayesian framework using the GeMTC package in R (version 3.4.1). The data synthesis was assumed to be feasible if clinical and methodological heterogeneity were negligible. We used relative risk (RR) values and 95% confidence intervals (CIs) as approximations to measure all-cause mortality. Mean differences (MDs) and 95% CIs were used to express the pooled differences in the durations of intensive care unit (ICU) stay, mechanical ventilation, and hospital stay. Statistical heterogeneity was assessed with the I^2^ statistic using the Higgins-Thompson method as follows: low heterogeneity, 25%; moderate heterogeneity, 50%; and high heterogeneity, 75%. Additionally, clinical heterogeneity was assessed based on the clinical characteristics presented in a clear study table. We chose the random-effects model because of the lower heterogeneity of the included studies, and we ranked the different types of immunotherapies based on outcome. The risk of bias of the included trials was assessed by two authors independently using the Cochrane risk of bias tool.

## Results

### Study identification

**Fig. 1:**
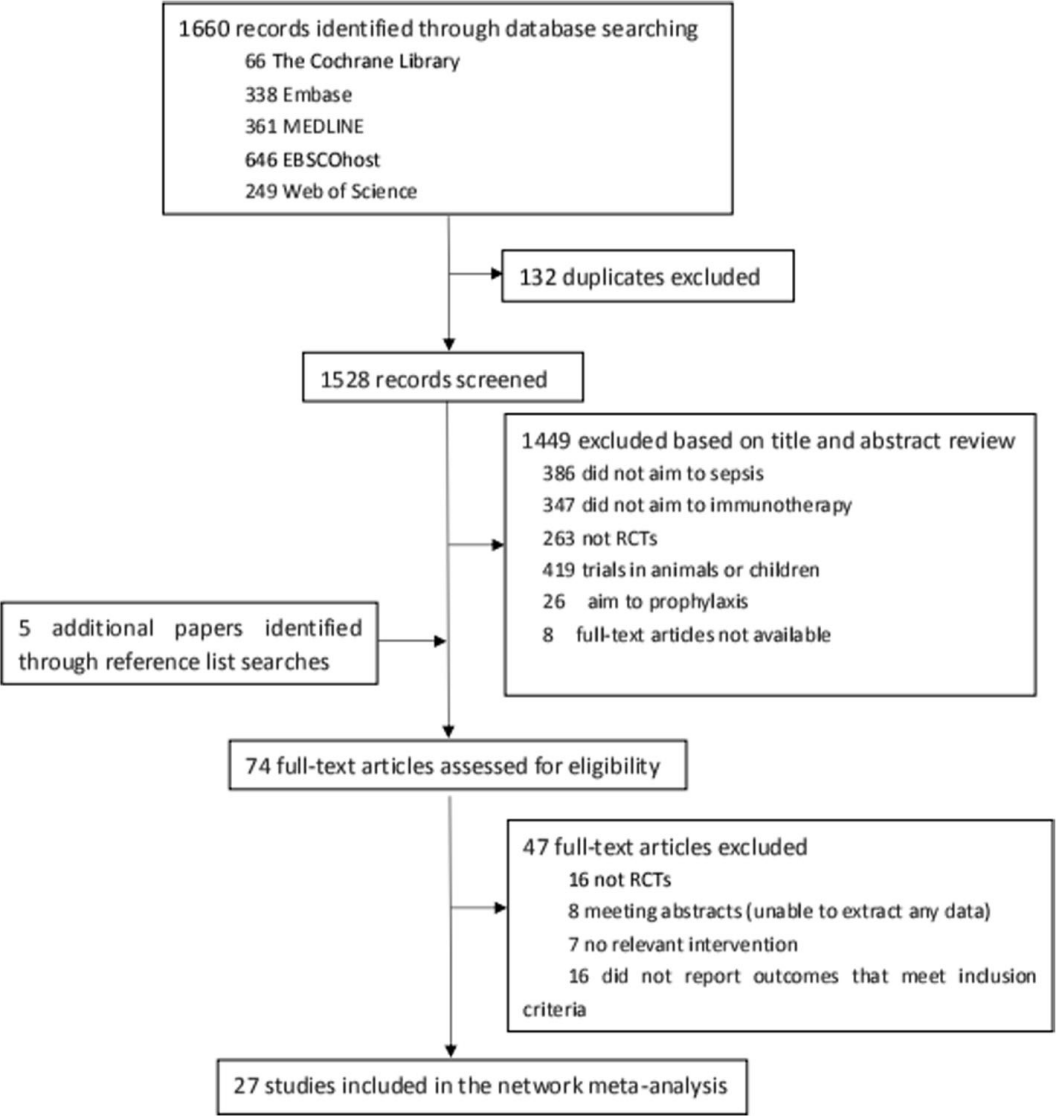
Flow diagram of the literature search

We identified 1,660 studies based on titles and abstracts. Then we retrieved the full text of 74 potentially eligible articles for an assessment (Fig. 1). Ultimately, we excluded 47 irrelevant articles; thus, the network meta-analysis included 27 RCTs [17, 18, 27–51] published between 1981 and 2015 that compared 4 types of immunotherapies with placebo (Table 1). There were 12 studies including 258 patients in the G-CSF group and 3 studies including 57 patients in the GM-CSF group. The IgG group contained 10 studies and 2002 patients. In addition, there were 5 studies including 175 patients in the IgGAM group and 27 studies including 2380 patients in the placebo group.

**Table 1:** Characteristics of the randomized controlled trials of different immunotherapies for neonates with suspected or proven sepsis

First author of publication	Number of participants (n)	Participants			Interventions			Outcomes
		Treatment (male)	Control 1 (male)	Control 2 (male)	Treatment	Control 1	Control 2	
Ahmad (2002) [27]	28	10 (NA)	10 (NA)	8 (NA)	G-CSF 5 μg/kg/12 h, IV, 7 days or until ANC ≥ 10000 cells/mm3	GM-CSF 4 μg/kg/12 h, IV, 7 days or until ANC ≥ 10000 cells/mm3	Placebo	All-cause mortality; hospital stay duration
Ahmed (2006) [28]	60	30 (15)	30 (16)		IgG 500 mg/kg/d, IV, 3 days	No intervention		All-cause mortality; hospital stay duration
Akdag (2014) [29]	102	51 (30)	51 (31)		IgGAM 250 mg/kg/d, IV, 3 days	Placebo (saline)		All-cause mortality; hospital stay duration
Aktas (2015) [30]	56	33 (23)	23 (17)		G-CSF 10 μg/kg/d, until ANC ≥ 1.0 × 109/L	No intervention		All-cause mortality
Bedford Russell (2001) [31]	28	13 (3)	15 (4)		G-CSF 10 μg/kg/d, IV, maximum of 14 days	Placebo		All-cause mortality; mechanical ventilation days; ICU stay duration
Bilgin (2001) [32]	60	30 (17)	30 (18)		GM-CSF 5 μg/kg/d, IH, 7 days	No intervention		All-cause mortality; hospital stay duration
Borjianyazdi (2013) [33]	46	23 (12)	23 (14)		GCSF 10 μg/kg/d, IH, 5 days	Placebo (saline)		All-cause mortality; hospital stay duration
Chaudhuri (2012) [34]	78	39 (22)	39 (24)		GCSF 10 μg/kg/d, IV, 3 days	Placebo (5% dextrose)		All-cause mortality
Chen (1996) [35]	56	28 (NA)	28 (NA)		IgG 500 mg/kg, one dose	Placebo (0.9% sodium chloride)		All-cause mortality
Christensen (1991) [36]	22	11 (NA)	11 (NA)		IgG 750 mg/kg, IV, one dose	Placebo (albumin)		All-cause mortality
Drossou-Agakidou (1998) [37]	35	19 (NA)	16 (NA)		G-CSF 10 lg/kg/d, IH, 3 days	No intervention		All-cause mortality
Drossou-Agakidou (2002) [38]	56	17 (8)	19 (9)	20 (13)	GM-CSF 5 mcg/kg/d, IV, 4 days	G-CSF 10 mcg/kg/d, IV, 4 days	Placebo (saline)	All-cause mortality; hospital stay duration; mechanical ventilation days
El-Ganzoury (2012) [39]	60	30 (15)	30 (16)		G-CSF 10 μg/kg/d, IV, 3 days	No intervention		All-cause mortality; hospital stay duration; mechanical ventilation days
Erdem (1993) [40]	44	20 (NA)	24 (NA)		IgGAM 5 ml/kg/d, IV, 3 days	No intervention		All-cause mortality
Gathwala (2012) [18]	40	20 (11)	20 (13)		G-CSF 10 mg/kg/d, IV, 5 days	No intervention		All-cause mortality; hospital stay duration
Gunes (2006) [41]	88	33 (18)	33 (12)		IgG 500 mg/kg, IV	No intervention		All-cause mortality
Haque (1988) [42]	60	30 (20)	30 (24)		IgGAM 5 mL/kg/d, IV, 4 days	Placebo (10% dextrose)		All-cause mortality
Haque (1995) [43]	195	65 (NA)	65 (NA)	65 (NA)	IgG 250 mg/kg/d, IV, 4 days	IgGAM, pentaglobin 250 mg/kg/d, IV, 4 days	no intervention	All-cause mortality
INIS Collaborative Group (2011) [17]	3493	1759 (NA)	1734(NA)		IgG 500 mg/kg/48 h, IV, 2 doses	Placebo (saline/ albumin solution)		All-cause mortality; hospital stay duration
Kucukoduk (2002) [44]	40	20 (12)	20 (11)		G CSF 5 μg/kg/d, IV, 3 days	Placebo (physiological serum)		All-cause mortality; ICU stay duration
Mancilla-Ramirez (1992) [45]	37	19 (NA)	18 (NA)		IgG 500 mg/kg, IV, one dose	Placebo (maltose)		All-cause mortality
Miura (2000) [46]	44	22 (9)	22 (14)		G-CSF 10 μg/kg/d, IV, 3 days	Placebo		All-cause mortality
Samatha (1997) [47]	60	30 (NA)	30 (NA)		IgGAM 5 mL/kg/d, 3 days	No intervention		All-cause mortality; hospital stay duration
Schibler (1998) [48]	20	10 (6)	10 (7)		G-CSF 10 μg/kg/d, IV, 3 days	Placebo		All-cause mortality
Shenoi (1999) [49]	57	25 (18)	25 (20)	7 (5)	IgG 1 g/kg, IV, 3 days	Placebo (0.15% saline in 10% dextrose)	no intervention	All-cause mortality; hospital stay duration; mechanical ventilation days
Sidiropoulos (1981) [50]	82	41 (NA)	41 (NA)		IgG preterm infants: 0.5 g/d, 6 days; term infants: 1.0 g/d, 6 days	No intervention		All-cause mortality
Weisman (1992) [51]	31	14 (7)	17 (10)		IgG 500 mg/kg, IV	Placebo (albumin)		All-cause mortality; hospital stay duration

*IgG* immunoglobulin, *IgGAM* IgM-enriched immunoglobulin, *G-CSFs* granulocyte-colony stimulating factor, *GM-CSF* granulocyte-macrophage colony stimulating factor, *IVIg* intravenous immunoglobulin

Among the selected trials, the mean gestational age of the patients ranged from 24 to 42 weeks, and approximately half of the patients were male. The median duration of the immunotherapy treatment was 6 days (range: 1–14 days). The included patients were diagnosed with either suspected or proven sepsis. Twenty-seven trials presented information on trial sample size calculation for various clinical outcome indices based on statistical principles (all-cause mortality: 27 trials; duration of hospital stay: 11 trials) (Additional file 2: Figure S1, Additional file 3: Figure S2). The sample sizes of the trials varied widely (20–3,493 patients) and included 12 trials that enrolled fewer than 50 patients.

### Quality assessment

We assessed the methodological quality of all the eligible RCTs as having a low, high, or unclear risk of bias for each criterion using the Cochrane Collaboration’s risk of bias tool (Additional file 4: Figure S3, Additional file 5: Figure S4). Most studies had an unclear risk of bias due to the absence of detailed reporting. We identified 13 trials with an unclear risk of bias, 2 trials with a high risk of bias in sequence generation and 2 trials with a high risk of bias for allocation concealment. Most of the trials were judged to have a low risk of bias for the blinding of patients. In particular, 77.8% of the studies included blinding for outcome assessment, 85.2% included blinding for incomplete outcome data, and 40.7% included blinding for allocation concealment. A high risk of bias was identified in 5 trials for blinding of participants and personnel and in 4 trials for blinding of outcome assessment. A high risk of bias for incomplete outcome data was detected in 2 trials. One trial exhibited a high risk of reporting bias, and 2 trials had high risk of other forms of bias. Publication bias is always a concern, and our meta-analysis included all randomized controlled trials that met the inclusion criteria to minimize publication bias.

### Network meta-analysis

**Fig. 2:**
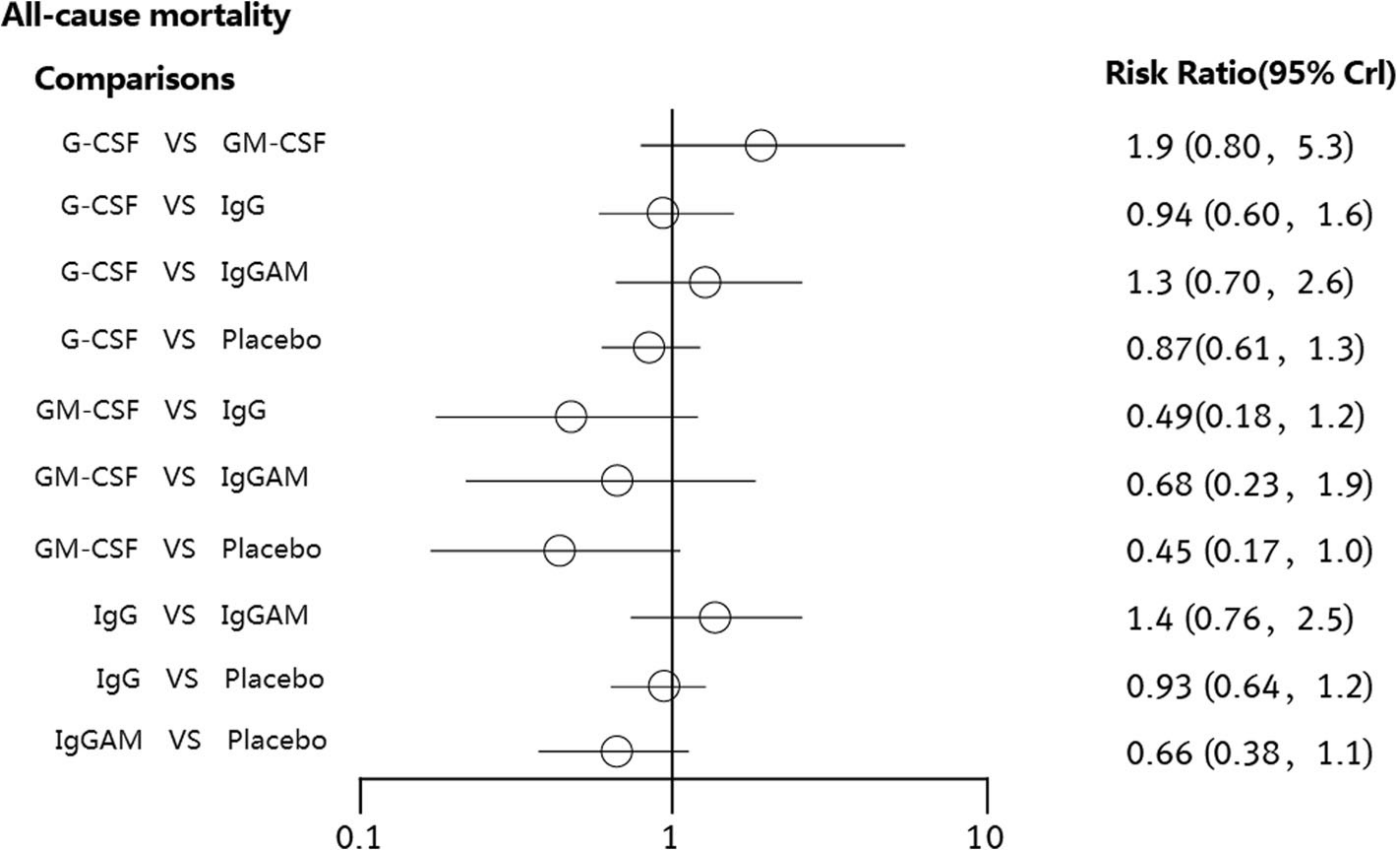
Risk ratios and 95% CIs for all-cause mortality in the five-node network meta-analysis

**Fig. 3:**
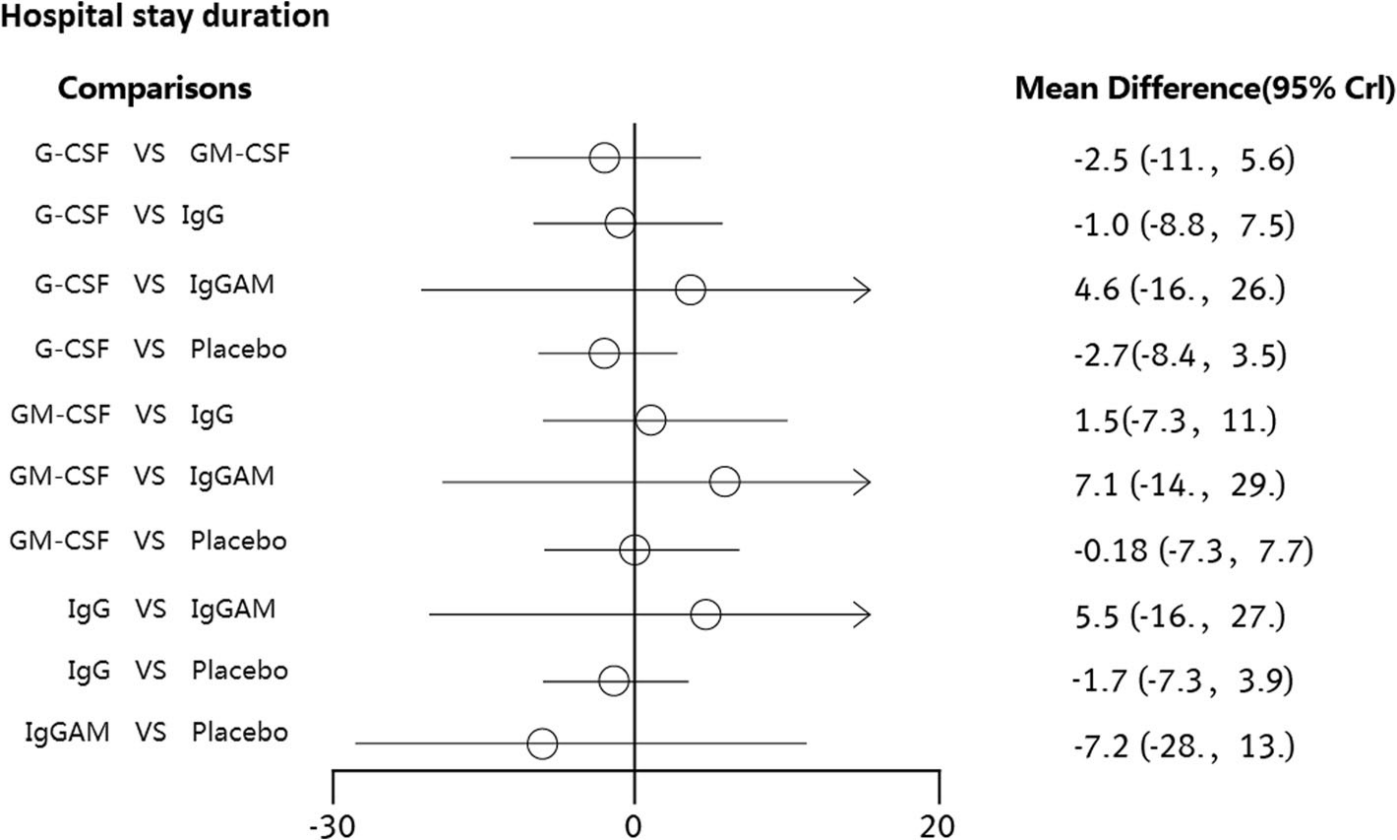
Risk ratios and 95% CIs for hospital stay in days in the five-node network meta-analysis

A total of 27 RCTs reported information on immunotherapies for neonates with sepsis. All the included studies reported data on all-cause mortality. We compared the effects of G-CSF, GM-CSF, IgG and IgGAM with the effect of placebo. The pooled effect sizes suggested that none of the interventions showed statistically significant differences from placebo. That is, these therapies were no more efficacious than placebo in reducing all-cause mortality (Fig. 2). The RRs of the immunotherapies G-CSF, GM-CSF, IgG and IgGAM were 0.80 (95% CI: 0.57 to 1.1), 0.45 (95% CI: 0.17 to 1.0), 0.93 (95% CI: 0.64 to 1.2) and 0.67 (95% CI: 0.39 to 1.1), respectively. The I^2^ values of placebo versus G-CSF, GM-CSF, IgG and IgGAM were 15.1, 0, 7.6 and 30.8%, respectively. A total of 14 RCTs reported information on the duration of hospital stay. We compared the effects of G-CSF, GM-CSF, IgG and IgGAM with the effect of placebo; however, compared with placebo, none of the interventions showed statistically significant differences (Fig. 3). The MDs of the immunotherapies were − 2.7 (95% CI: − 8.4 to 3.5), − 0.18 (95% CI: − 7.3 to 7.7), − 1.7 (95% CI: − 7.3 to 3.9) and − 7.2 (95% CI: − 28 to 13), respectively. The I^2^ values of placebo versus G-CSF, GM-CSF, IgG and IgGAM were 83.6, 0, 13.2 and 0%, respectively. Because the results of the experiments included in the present study were incomplete, the durations of mechanical ventilation and ICU stay were not evaluated. Additional file 6: Figure S5 and Additional file 7: Table S1 summarize the rankings of the different immunotherapies based on all-cause mortality. For probability ranking, GM-CSF immunotherapy exhibited the greatest potential for reducing mortality, and the probability of GM-CSF having the top ranking was 76.2%. Placebo was estimated to be the worst therapy in terms of all-cause mortality.

## Discussion

Over the past few decades, various trials have investigated the effects of immunotherapies on neonates with sepsis. However, no previous meta-analysis has compared the effects of different types of immunotherapies with the effect of placebo. Each of these previous studies compared only a single type of immunotherapy with a placebo. Our five-node meta-analysis represents the most comprehensive synthesis of current data on immunotherapies for neonates with sepsis. We found that immunotherapy was not significantly more efficacious than placebo.

Ohlsson’s meta-analysis reported that mortality was not significantly different after the use of intravenous immunoglobulin therapy for suspected or proven infection in neonates. The same author updated meta-analyses on the use of intravenous immunoglobulin for suspected or proven infection in neonates [52–54]. In the most recent meta-analysis published by Ohlsson’s group in 2015, 9 studies with a total of 3,973 infants included data on the outcome of all-cause mortality, and 3 studies with a total of 170 infants included data on the outcome of hospital stay duration [54]. In Franco’s meta-analysis, the mortality rate was evaluated for 7 RCTs including 3,756 patients. The global effect of intravenous immunoglobulin vs placebo treatment on mortality was not significantly different [20]. In the present study, we included 14 studies on the use of intravenous immunoglobulin; however, the updated studies exhibited similar results. Kreymann et al. analyzed the effects of intravenous polyclonal immunoglobulins on mortality and other clinical outcomes. Their results indicated that polyvalent immunoglobulins exert a significant effect on mortality in sepsis and septic shock, with a trend toward immunoglobulin enrichment with IgA and IgM [19]. However, their study differed from ours. They included neonates from 24 weeks to 42 weeks gestational age, and there are likely huge differences in the immune response capabilities across such an extreme age range that may have notably influenced the results. In addition, some new randomized controlled trials were included in our meta-analysis, but not in theirs. Furthermore, our study was a network meta-analysis that enabled a variety of immunotherapies to be compared together.

We included 12 trials on G-CSF therapies that reported all-cause mortality and compared their outcomes with those of other immunotherapies. Another meta-analysis also reported similar results: In this meta-analysis, conducted by Bernstein, 5 trials with a total of 155 patients were evaluated, and mortality was lower among the G-CSF recipients than among the placebo recipients. However, when nonrandomized studies were excluded, the beneficial effects of G-CSF therapy tended to be less consistent [55]. Thus, routine use of G-CSF cannot be recommended for all neonates with sepsis.

Many patient-related factors, such as an underlying state of immunosuppression, the time between sepsis diagnosis and immunotherapy administration, and concurrent treatments, can influence the clinical effects of immunotherapy. Due to individual patient-specific situations, such as the neonates’ immunological state, some types of immunotherapy might not elicit a significant mortality benefit. Many reports also suggest that the time to the initiation of clinical support measures, particularly antibiotic therapy, control of the infection source, and potential hemodynamic support, can influence trial outcomes. A previous meta-analysis by Busani et al. showed that the immunoglobulin composition was not a significant source of heterogeneity in their subgroup analysis, and they found that studies that used IgGAM showed a more consistent reduction in mortality in the treatment arm than studies using polyclonal immunoglobulins. Different dosing regimens and treatment durations also appeared to affect the results [56]. However, the optimal dose, duration, and composition of the interventions remain unclear. Thus, considering the large variations in immune responses neonates as well as the interactions between proinflammatory processes and immune defects during the onset and later phases of development, comprehensive stratification of neonatal sepsis is of vital importance. Additional studies are necessary to optimize interventions for sepsis patients.

Our understanding of immunology and the pathophysiological basis of neonatal sepsis is still developing, and the mechanisms of immunomodulation in sepsis remain unclear. Additional high-quality studies are therefore required to identify the immunomodulation mechanisms in the different phases of sepsis. Therapies aimed at treating neonatal sepsis must also consider their unique immunological status.

### Limitations

Our study has several limitations. First, all-cause mortality alone may not be a valid endpoint; evaluation of short-term or long-term mortality may provide additional information. Thus, additional RCTs that report short-term or long-term mortality are required. Moreover, a consensus needs to be reached if progress is to be made in the development of efficacious immunotherapies for neonatal sepsis. Second, because the results of the experiments included in the present study are incomplete, network meta-analyses of the durations of mechanical ventilation and ICU stay were not conducted. Therefore, the results of our study are relatively simple, and more comprehensive and diverse conclusions could not be provided. Third, head-to-head trials assessing the efficacy of different immunotherapies for neonates with sepsis are limited, and the network meta-analysis may rely heavily on indirect comparisons. Thus, the output of the network meta-analysis is prone to false negative or false positive results. Fourth, the studies included in our meta-analyses were conducted over a wide time range, during which the definition of neonatal sepsis and the methods for diagnosis and treatment changed; consequently, patients diagnosed with suspected sepsis may be receiving immunotherapies that are not actually be treating an infectious process. Additionally, only one study (Brocklehurst et al.) was a multicenter study; it included a population of 3,493 patients, which was much larger than the sample sizes of the other studies [17]. Finally, some of the effects of immunotherapy could not be analyzed in detail as they were reported in only a small number of RCTs: there were 12 studies in the G-CSF group, 3 studies in the GM-CSF group, 10 studies in the IgG group and 5 studies in the IgGAM group. Future multicenter studies involving larger sample sizes and direct parallel comparisons among different therapies are needed to confirm our results.

## Conclusion

We found that compared with placebo, immunotherapy does not elicit a significant difference in all-cause mortality or the duration of hospital stay in neonates with suspected or proven sepsis.

## Additional files

Additional file 1: Text E1 Search strategies. (DOCX 14 kb)

Additional file 2: **Figure S1.** Network of the comparisons for the five-node network meta-analysis on all-cause mortality. (DOCX 60 kb)

Additional file 3: **Figure S2.** Network of the comparisons for the five-node network meta-analysis on hospital stay duration. (DOCX 51 kb)

Additional file 4: **Figure S3.** Risk of bias graph. (DOCX 19 kb)

Additional file 5: **Figure S4.** Risk of bias summary. (DOCX 28 kb)

Additional file 6: **Figure S5.** Possibility ranking based on simulations in terms of all-cause mortality in the five-node network meta-analysis. (DOCX 27 kb)

Additional file 7: **Table S1.** Possibility ranking based on simulations in terms of all-cause mortality in the five-node network meta-analysis. (DOCX 12 kb)

## Data Availability

Supporting data can be obtained from the corresponding author.

## Abbreviations

APC: Antigen-presenting cell
CI: Confidence interval
G-CSF: Granulocyte-colony stimulating factor
GM-CSF: Granulocyte-macrophage colony stimulating factor
ICU: Intensive care unit
IgG: Immunoglobulin
IgGAM: IgM-enriched immunoglobulin
IVIg: Intravenous immunoglobulin
MD: Mean difference
RCT: Randomized controlled trial
RR: Relative risk
