# Supplementary material for: Effects of immunotherapy on mortality in neonates with suspected or proven sepsis: a systematic review and network meta-analysis
Source: BMC Pediatr. 2019 Aug 5;19:270. doi: 10.1186/s12887-019-1609-1 (PMC6681492; doi:10.1186/s12887-019-1609-1)
Supplement: Supplementary file 1 — Text E1 Search strategies. (DOCX 14 kb) [file 12887_2019_1609_MOESM1_ESM.docx]

Additional file 1: Text E1 :

Search strategies

1.Search strategy for CENTRAL, The Cochrane Library (66)

#1 MeSH descriptor: [Immunoglobulins] explode all trees

#2 MeSH descriptor: [Immunoglobulins, Intravenous] explode all trees

#3 MeSH descriptor: [Granulocyte Colony-Stimulating Factor] explode all trees

#4 MeSH descriptor: [Granulocyte-Macrophage Colony-Stimulating Factor] explode all trees

#5  "immunoglobulin*" or IVIG* or "Granulocyte Colony-Stimulating Factor*" or GCSF* or "Granulocyte Colony-Stimulating Factor, recombinant" or "Granulocyte-Macrophage Colony-Stimulating Factor*" or GMCSF* or "Granulocyte-Macrophage Colony-Stimulating Factor，recombinant" ti,ab,kw

#6 #1 or #2 or #3 or #4 or #5

#7 MeSH descriptor: [Sepsis] explode all trees

#8 MeSH descriptor: [Shock, Septic] explode all trees

#9 #7 or #8

#10 MeSH descriptor: [Randomized Controlled Trials as Topic] explode all trees

#11 ((randomized controlled trial or controlled clinical trial) .pt. or randomized.ab. or placebo.ab. or clinical trials as topic.sh. or randomly.ab. or trial.ti.) not (animals not (humans and animals))

#12 #10 or #11

#13 MeSH descriptor infant explode all trees

#14 MeSH descriptor newborn explode all trees

#15 MeSH descriptor neonate explode all trees

#16 "infant" or "newborn" or "neonate"

#17 #13 or #14 or #15 or #16

#18 #6 and #9 and #12 and #17

1. Search strategy for EMBASE (338)
2. immunoglobulins* OR (immunoglobulins* AND intravenous*) OR 'granulocyte colony stimulating factor*' OR 'granulocyte macrophage colony stimulating factor*' OR immunoglobulin* OR ivig* OR 'granulocyte colony stimulating factor*' OR gcsf* OR 'recombinant granulocyte colony stimulating factor' OR 'recombinant granulocyte colony stimulating factor*' OR 'granulocyte macrophage colony stimulating factor' OR 'granulocyte macrophage colony stimulating factor*' OR gmcsf* OR 'recombinant granulocyte macrophage colony stimulating factor' OR 'recombinant granulocyte macrophage colony stimulating factor*'

2. 'sepsis' OR 'sepsis*' OR 'septic shock' OR 'septic shock*'

3. 'infant' OR 'newborn' OR 'neonate'

4. 1 and 2 and 3

5. 'randomized controlled trial' OR 'controlled clinical trial' OR randomized OR 'placebo' OR 'clinical trial (topic)' OR randomly OR trial OR 'clinical trial' OR ((clinical AND trials AND as AND topic OR randomly OR trial) NOT (animals NOT (humans AND animals)))

6. 4 and 5

1. Search strategy for MEDLINE (361)

1. exp sepsis /or sepsis*/ or septic shock /or septic shock*.mp.

2. exp immunoglobulins or immunoglobulins, Intravenous or immunoglobulin* or IVIG* or Granulocyte Colony-Stimulating Factor / or Granulocyte Colony-Stimulating Factor* or GSCF* or Granulocyte Colony-Stimulating Factor recombinant or Granulocyte-Macrophage Colony-Stimulating Factor / or Granulocyte-Macrophage Colony-Stimulating Factor* or GMCSF* or Granulocyte-Macrophage Colony-Stimulating Factor recombinant .mp.

3.1 and 2

4. ((randomized controlled trial or controlled clinical trial).pt. or randomized.ab. or placebo.ab. or clinical trials as topic.sh. or randomly.ab. or trial.ti.) not (animals not (humans and animals)).sh.

5. 3 and 4

4. Search strategy for CINAHL (EBSCOhost) (646)

S1 (MH ”Immunoglobulins”) OR (MH “Immunoglobulins, Intravenous”) OR (MH “Granulocyte Colony-Stimulating Factor”) OR (MH “Granulocyte-Macrophage Colony-Stimulating Factor”) OR "immunoglobulin*" OR IVIG* OR "Granulocyte Colony-Stimulating Factor*" OR GCSF* OR "Granulocyte Colony-Stimulating Factor, recombinant" OR "Granulocyte-Macrophage Colony-Stimulating Factor*" OR GMCSF* OR "Granulocyte-Macrophage Colony-Stimulating Factor, recombinant"

S2 (MH “Sepsis”) OR (MH “Shock, Septic”)

S3 ((MH “Randomized Controlled Trial as Topic”) OR ((randomized controlled trial or controlled clinical trial) or randomized or placebo or clinical trials as topic or randomly or trial) not (animals not (humans and animals))

S4 (MH “[Infant, Newborn](https://www.ncbi.nlm.nih.gov/mesh/68007231)”) OR (MH “Neonate”)

S5 S1 and S2 and S3 and S4

5. Search strategy for ISIWeb of Science (249)

#1 TS=Immunoglobulins* or Immunoglobulins, Intravenous* or Granulocyte Colony-Stimulating Factor* or Granulocyte-Macrophage Colony-Stimulating Factor* or Immunoglobulin* or GCSF* or Granulocyte Colony-Stimulating Factor, recombinant* or GMCSF* or Granulocyte-Macrophage Colony-Stimulating Factor, recombinant*

#2 TS=(sepsis* or shock,septic)

#3 TS=(infant or newborn or neonate)

#4 TS=((randomized controlled trial or controlled clinical trial or randomized or placebo or clinical trials as topic or randomly or trial) not (animals not (humans and animals)))

#5 #1 and #2 and #3 and #4
