# Supplementary material for: Effects of immunotherapy on mortality in neonates with suspected or proven sepsis: a systematic review and network meta-analysis
Source: BMC Pediatr. 2019 Aug 5;19:270. doi: 10.1186/s12887-019-1609-1 (PMC6681492; doi:10.1186/s12887-019-1609-1)

Additional file 3: Figure S2:

Network of the comparisons for the five-node network meta-analysis on hospital stay duration

The size of the nodes is proportional to the number of patients (in parentheses) randomly assigned to receive the treatment. The width of the lines is proportional to the number of trials (next to the line) that compare the connected treatments. A. Granulocyte-colony stimulating factor (G-CSF); B. Granulocyte-macrophage colony stimulating factor (GM-CSF); C. Immunoglobulin (IgG); D. IgM-enriched immunoglobulin (IgGAM); E. Placebo.


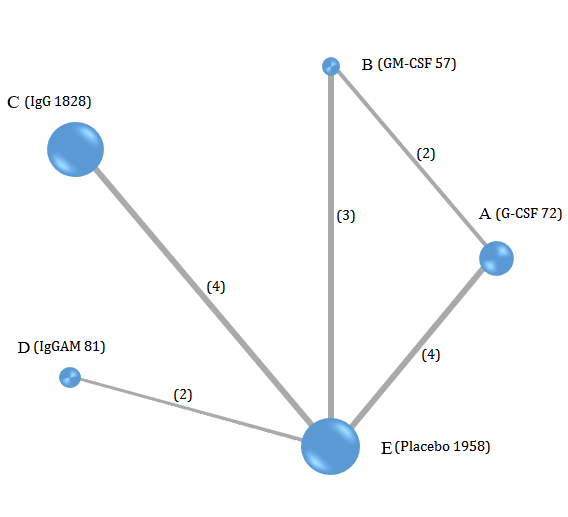

Supplement: Supplementary file 3 — Figure S2. Network of the comparisons for the five-node network meta-analysis on hospital stay duration. (DOCX 51 kb) [file 12887_2019_1609_MOESM3_ESM.docx]
