# Supplementary material for: Effects of immunotherapy on mortality in neonates with suspected or proven sepsis: a systematic review and network meta-analysis
Source: BMC Pediatr. 2019 Aug 5;19:270. doi: 10.1186/s12887-019-1609-1 (PMC6681492; doi:10.1186/s12887-019-1609-1)

Additional file 5: Figure S4:

Risk of bias summary.

Review authors' judgements about each risk of bias item of all included studies. Red=high risk; green=low risk; yellow=unclear.


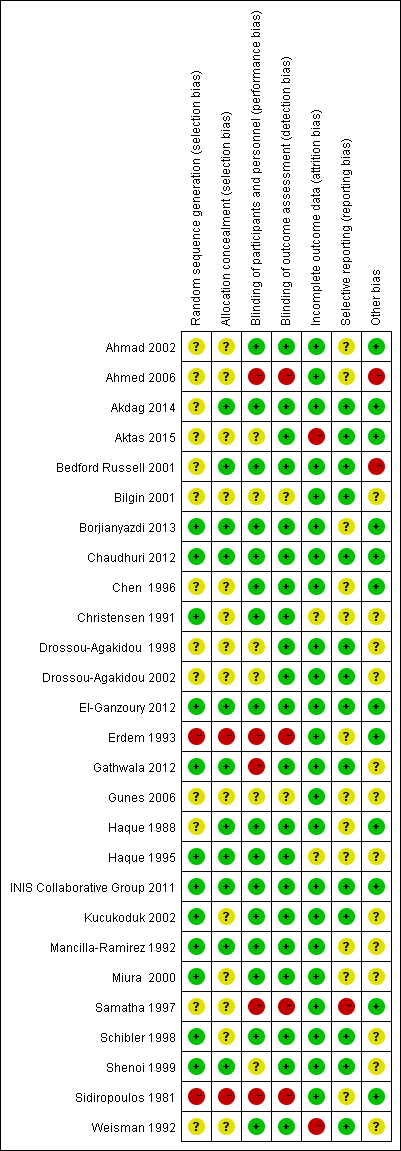

Supplement: Supplementary file 5 — Figure S4. Risk of bias summary. (DOCX 28 kb) [file 12887_2019_1609_MOESM5_ESM.docx]
