# Supplementary material for: Effects of immunotherapy on mortality in neonates with suspected or proven sepsis: a systematic review and network meta-analysis
Source: BMC Pediatr. 2019 Aug 5;19:270. doi: 10.1186/s12887-019-1609-1 (PMC6681492; doi:10.1186/s12887-019-1609-1)

Additional file 6: Figure S5:

Possibility ranking based on simulations in terms of all-cause mortality in the five-node network meta-analysis

A. Granulocyte-colony stimulating factor (G-CSF); B. Granulocyte-macrophage colony stimulating factor (GM-CSF); C. Immunoglobulin (IgG); D. IgM-enriched immunoglobulin (IgGAM); E. Placebo.


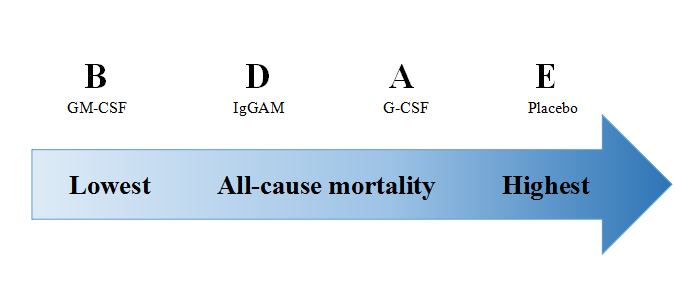

Supplement: Supplementary file 6 — Figure S5. Possibility ranking based on simulations in terms of all-cause mortality in the five-node network meta-analysis. (DOCX 27 kb) [file 12887_2019_1609_MOESM6_ESM.docx]
