# Supplementary material for: Effects of immunotherapy on mortality in neonates with suspected or proven sepsis: a systematic review and network meta-analysis
Source: BMC Pediatr. 2019 Aug 5;19:270. doi: 10.1186/s12887-019-1609-1 (PMC6681492; doi:10.1186/s12887-019-1609-1)
Supplement: Supplementary file 7 — Table S1. Possibility ranking based on simulations in terms of all-cause mortality in the five-node network meta-analysis. (DOCX 12 kb) [file 12887_2019_1609_MOESM7_ESM.docx]

Additional file 7: Table S1:

Possibility ranking based on simulations in terms of all-cause mortality in the five-node network meta-analysis.

|  | [,1] | [,2] | [,3] | [,4] | [,5] |
| --- | --- | --- | --- | --- | --- |
| A | 0.177660 | 0.209795 | **0.413700** | 0.17859 | 0.020255 |
| B | 0.023780 | 0.017660 | 0.040720 | 0.15600 | **0.761840** |
| C | 0.248770 | 0.333265 | 0.306055 | 0.09936 | 0.012550 |
| D | 0.043585 | 0.049745 | 0.143940 | **0.55764** | 0.205090 |
| E | **0.506205** | **0.389535** | 0.095585 | 0.00841 | 0.000265 |
